# Development of dedicated phantom for end‐to‐end testing of multiple image‐guidance configurations incorporating light‐section‐based surface guidance system

**DOI:** 10.1002/acm2.14517

**Published:** 2024-11-26

**Authors:** Naoki Hayashi, Tatsunori Saito, Kazuki Ouchi, Hiroshi Amma, Yuta Muraki, Keisuke Yasui, Masashi Nozue

**Affiliations:** ^1^ Division of Medical Physics School of Medical Sciences Fujita Health University Toyoake Aichi Japan; ^2^ Department of Radiology Seirei Hamamatsu General Hospital Hamamatsu Shizuoka Japan; ^3^ Department of Radiology Japanese Red Cross Aichi Medical Center Nagoya Daiichi Hospital Nagoya Japan; ^4^ Department of Radiation Oncology Seirei Hamamatsu General Hospital Hamamatsu Shizuoka Japan

**Keywords:** coordinate coincidence, end‐to‐end testing, light‐section method, quality assurance, surface‐guided radiation therapy

## Abstract

**Purpose:**

Radiotherapy devices with multiple image‐guidance systems, such as surface‐guided radiation therapy (SGRT), have been widely used in recent years. However, in the case of SGRT devices using the light‐section method, coordinate coincidence evaluation using a phantom for SGRT devices with patterned light projection is not appropriate. Hence, this study aims to develop a dedicated phantom able to evaluate both the detection accuracy and coordinate coincidence of multiple IGRT configurations, including light‐section‐based SGRT devices.

**Materials and method:**

First, we developed an end‐to‐end (E2E) phantom that can be scanned by CT and detected by a light‐section‐method‐based SGRT device. Second, the detection accuracy of the phantom under three reference data acquisition conditions was evaluated using the E2E phantom. The three conditions were a body surface image detected by VOXELAN in the simulation CT room, a body surface image reconstructed from the volume data of the simulation CT room, and a body surface image acquired by VOXELAN in the radiotherapy room. Finally, the coordinate coincidence of the image‐guidance system was evaluated using the E2E phantom.

**Result:**

Upon comparing detection accuracy among the three reference data acquisition methods, we found that the reference data generated in the CT room had the largest error (0.58 mm at maximum). The coordinate coincidences of the multiple image‐guidance systems were within 1 mm for all components after the maintenance of VOXELAN using E2E phantom. Furthermore, the long‐term direction stability was worse in the longitudinal direction.

**Conclusion:**

The new E2E phantom can be used to evaluate the detection accuracy of a light‐section‐based SGRT system and the coordinate coincidence using a Winston–Lutz‐based method in multiple image‐guided configurations. The detection accuracy in the three different reference images of VOXELAN using this phantom improved to within 1 mm in all directions.

## 1 INTRODUCTION

Image‐guided radiation therapy (IGRT) is an advanced technique used in radiation oncology to enhance the precision and accuracy of radiation therapy delivery to target volumes.^1^, ^2^, ^3^ The primary advantage of IGRT is its ability to improve treatment outcomes while minimizing damage to normal tissues.^4^, ^5^, ^6^ IGRT enables real‐time visualization of the tumor and surrounding organs, allowing for more precise targeting of the radiation beams.^7^ This accuracy helps in the precise delivery of radiation to the tumor while sparing nearby normal tissues. Furthermore, IGRT can be used to monitor the response of tumors to treatment. If the tumor size or shape changes during treatment, adjustments can be made in real time to ensure optimal therapeutic effects. Surface‐guided radiation therapy (SGRT) has recently been introduced into IGRT workflow. SGRT is an advanced radiation therapy technique that utilizes real‐time imaging and tracking of the patient's skin surface to ensure accurate radiation treatment delivery. This involves using a specialized camera system and three‐dimensional (3D) surface‐imaging technology to monitor the body position and movement of the patient during treatment sessions.^8^ The combination of SGRT and X‐ray‐based IGRT enables patient localization and monitoring during irradiation. Furthermore, a reduced patient exposure dose is achieved compared with that achieved with X‐ray‐based IGRT alone.^9^, ^10^, ^11^, ^12^, ^13^ Therefore, radiotherapy machines with both (internal and external) image guidance have been employed in recent years.^14^, ^15^ In such cases, the coordinates of the SGRT device in combination with IGRT device must coincide with those provided by IGRT (a two‐dimensional (2D) X‐ray image and cone‐beam computed tomography (CBCT)).^16^ In addition, it is important to evaluate coordinate coincidence using dedicated phantoms and the Winston–Lutz test for periodic quality assurance (QA), as discussed in various studies.^17^, ^18^

**FIGURE 1:**
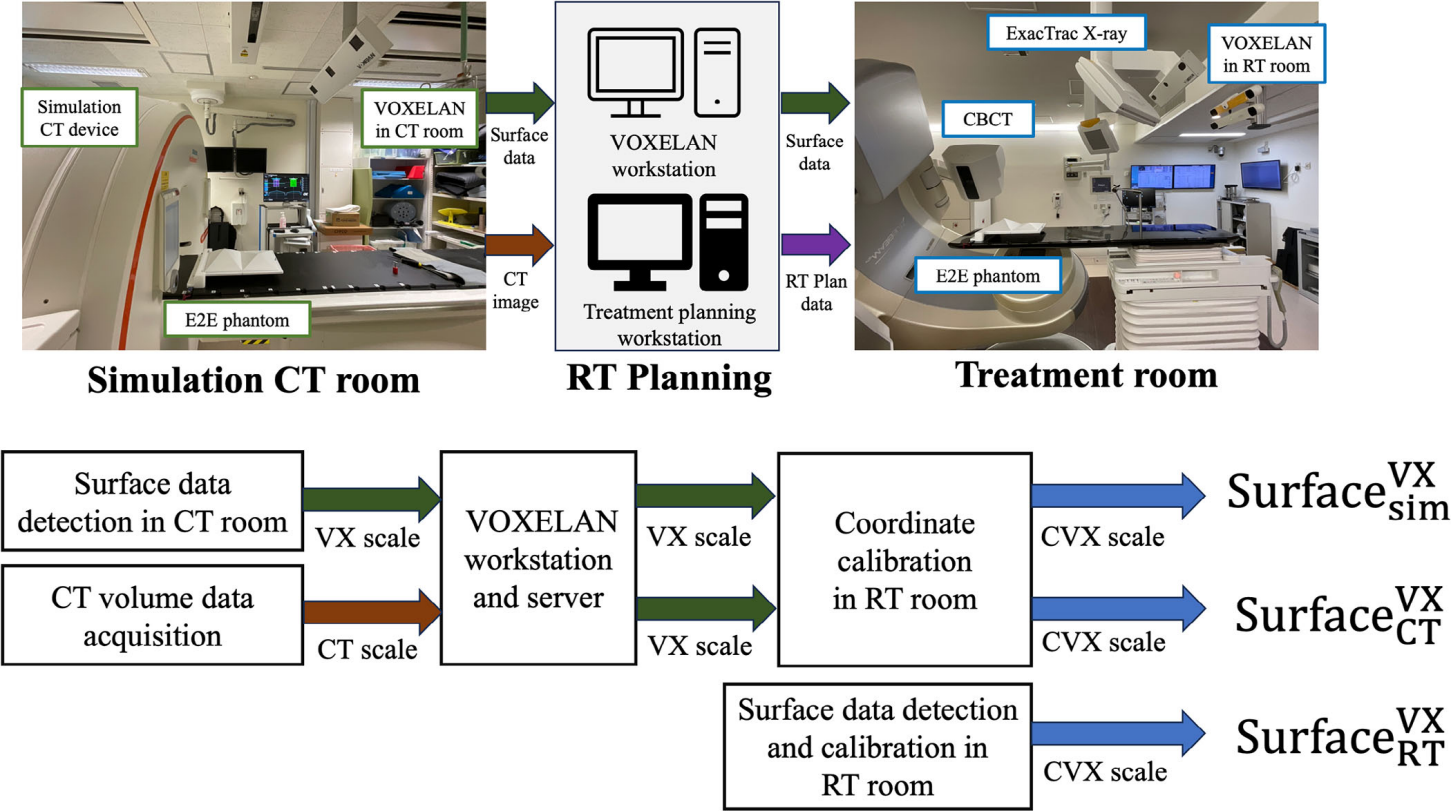
Data flow and types of surface data for radiotherapy using VOXELAN. VOXELAN systems were installed in the simulation CT room and radiotherapy treatment room in Seirei Hamamatsu General hospital. Therefore, we can scan the patient's surface data in both the simulation CT room and treatment room using VOXELAN. In addition, surface data can be developed by reconstructing planning CT images. The surface data in the CT room can be calibrated for the RT room. CT scale, computed tomography coordinate scale; CVX scale, calibrated VOXELAN coordinate scale; VX scale, VOXELAN coordinate scale.

In Seirei Hamamatsu General hospital, a radiotherapy system (TrueBeam STx, Varian, USA) equipped with ExacTrac X‐ray (ETX: BrainLAB, Feldkirchen, Germany) and VOXELAN (ERD Corporation, Okayama, Japan) is used for 2D X‐ray imaging and SGRT, respectively. VOXELAN is a light‐section‐based body‐surface monitoring device that differs from other SGRT devices. In VOXELAN, two slit lasers scan the patient's body, and the red slit laser form is detected using a charge‐coupled camera to determine the shape of the surface. VOXELAN can be attached to any radiotherapy device and used simultaneously with several image‐guidance devices. Furthermore, body surface data from planning computed tomography (CT) scans can be reconstructed using VOXELAN and used as reference data, or the reference data can be acquired from the radiation therapy room. In addition, patient surface data can be obtained during simulation CT by installing a VOXELAN in the simulation CT room (Figure 1). In this configuration, the VOXELAN in the radiotherapy treatment (RT) room allows to be able to use three reference surface datasets. In other words, a body surface image detected using VOXELAN in the simulation CT room (${\mathrm{Surface}}_{\mathrm{sim}}^{\mathrm{VX}}$), a body surface image reconstructed from the volume data of the simulation CT (${\mathrm{Surface}}_{\mathrm{CT}}^{\mathrm{VX}}$), and a body surface image acquired using VOXELAN in the RT room (${\mathrm{Surface}}_{\mathrm{RT}}^{\mathrm{VX}}$). Thus, for the device that allows multiple methods and methods for acquiring reference data, it is necessary to evaluate the coordinate coincidence for each condition. In this situation, an end‐to‐end (E2E) test is reasonable to evaluate spatial uncertainty during the entire process.^19^, ^20^ However, unlike other SGRT systems, VOXELAN uses a light‐section method to scan objects.^21^ There is no appropriate quality assurance phantom for the light‐section method. Therefore, it is necessary to develop a phantom tailored for multiple IGRT configurations using a light‐section‐based SGRT device.

This study aims to develop a dedicated phantom that can evaluate both the detection accuracy and coordinate coincidence of multiple IGRT configurations, including a light‐section‐based SGRT device.

## 2 MATERIALS AND METHODS

### 2.1 Phantom design

**FIGURE 2:**
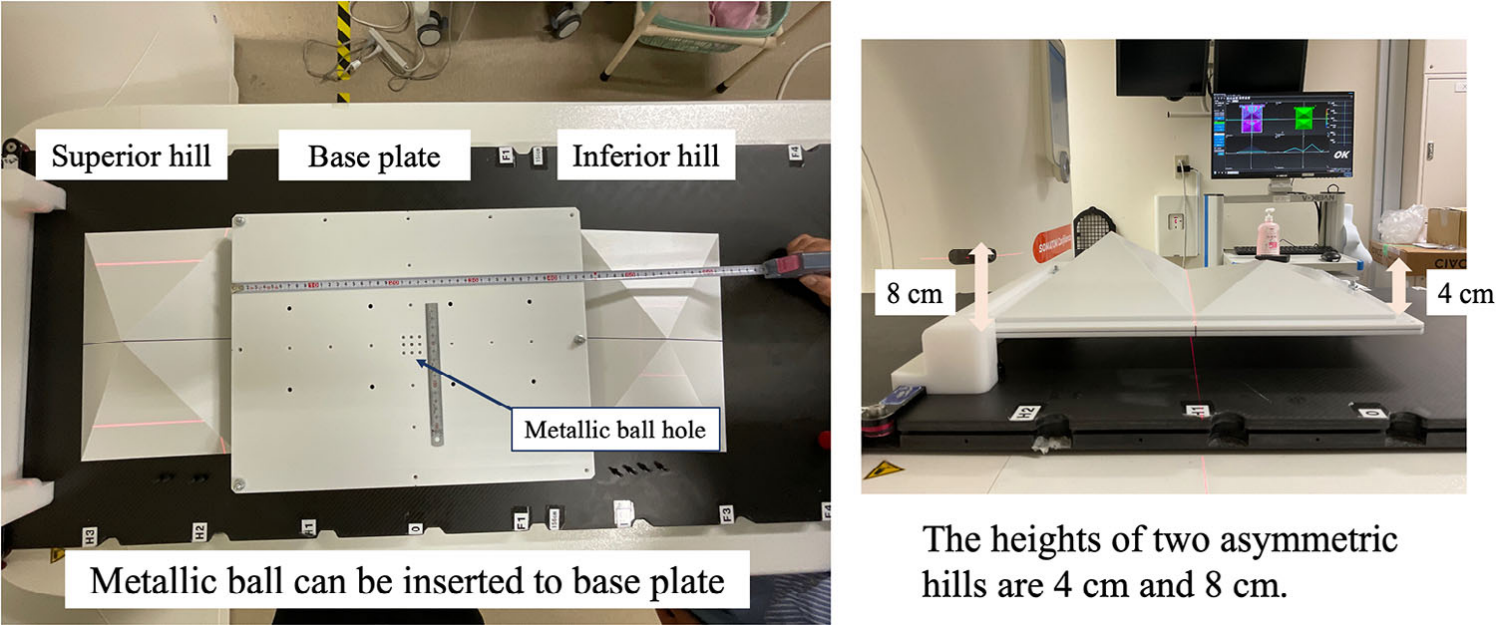
Phantom design for end‐to‐end testing. The dedicated phantom was designed with two hills on the base plate. The heights of the hills were selected as 4 or 8 cm. Metallic balls can be inserted into the metallic holes on the base plate. Metallic holes were spaced 5 mm apart in the center of the base plate and 5 cm apart in the peripheral area. This phantom was fabricated from white polyethylene terephthalate. The sizes of the phantom in the longitudinal, transverse, and height directions were 540, 390, and 110 mm, respectively.

A dedicated phantom was developed to perform E2E in an IGRT configuration, which included 2D X‐ray image, CBCT, and a light‐section‐based SGRT device (VOXELAN). The concept of the phantom design was to ensure reliable detection and easy setup of all IGRT devices. In previously published reports, VOXELAN has reliably detected asymmetric objects with distinctive shapes.^22^ In addition, white objects reliably and effectively reflect red lasers. Therefore, we designed a phantom with two hills of different heights, as illustrated in Figure 2. This phantom was designed to evaluate coordinate coincidence in IGRT configurations, including light‐section‐based SGRT devices. We termed this phantom the E2E phantom for light‐section‐based SGRT device. From now on, this phantom is described as E2E phantom in this paper for the simplicity. We made it possible to insert small metallic balls with a diameter of 5 mm into the phantom to perform the Winston–Lutz test‐based E2E.^23^ The heights of the asymmetric hills were either 4 or 8 cm. Heights of 4 and 8 cm were selected because they represent the minimum and maximum heights, respectively, in the range of heights detectable by VOXELAN. Furthermore, the greater the difference in height between the two hills of the phantom, the better the structure for VOXELAN to be detected. The more features the structure has, the less likely VOXELAN is to produce detection errors.

### 2.2 Comparison of detection accuracy in different reference image acquisition methods

In an environment where VOXELAN is installed in both the CT and RT rooms, reference images can be acquired using three methods: a body surface image detected by VOXELAN in the simulation CT room (${\mathrm{Surface}}_{\mathrm{sim}}^{\mathrm{VX}}$), a body surface image reconstructed from the volume data of the simulation CT (${\mathrm{Surface}}_{\mathrm{CT}}^{\mathrm{VX}}$), and a body surface image acquired by VOXELAN in the RT room (${\mathrm{Surface}}_{\mathrm{RT}}^{\mathrm{VX}}$). The detection accuracies of the E2E phantom in the RT room for the three reference image‐acquisition methods were compared. First, a reference image of the E2E phantom at the coordinate origin of the CT was acquired using VOXELAN in the CT room when a CT scan for the E2E phantom was performed (${\mathrm{Surface}}_{\mathrm{sim}}^{\mathrm{VX}}$). Next, the CT image of the E2E phantom was transferred to the VOXELAN workstation, and a reference image reconstructed from the CT image was acquired (${\mathrm{Surface}}_{\mathrm{CT}}^{\mathrm{VX}}$). Finally, the E2E phantom was placed on the radiotherapy treatment couch and a reference image at the isocenter origin was acquired (${\mathrm{Surface}}_{\mathrm{RT}}^{\mathrm{VX}}$). To compare the detection accuracy in three acquisition method, E2E phantom was set on the treatment couch at the calibration point as references. Then the E2E phantom was scanned for 10 s using VOXELAN. VOXELAN requires 0.5 s to scan when the maximum region of interest (ROI) setting is used. Therefore, there were 21 samples of detection data in a 10‐s scanning period. Raw data were analyzed among three acquisition methods.

### 2.3 Evaluation of coordinate coincidence between multiple IGRT devices

**FIGURE 3:**
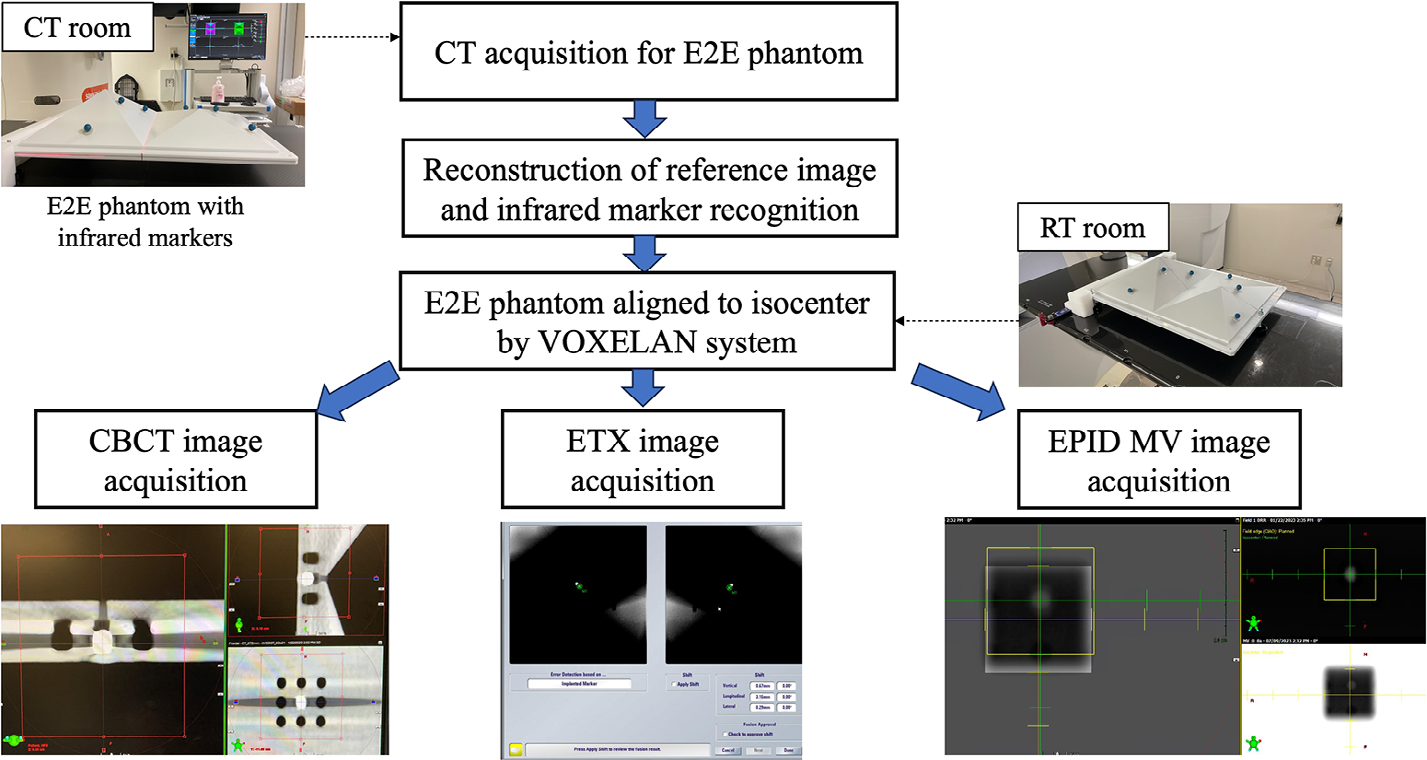
Procedure for evaluation of coordinate coincidences between multiple IGRT devices. E2E phantom with infrared markers was scanned using CT and reconstructed to create a reference image. VOXELAN in the room was used to align the E2E phantom to the isocenter using a reference image. Then, CBCT, ETX, and EPID MV images were acquired to evaluate the coordinate coincidence. CBCT, cone beam computed tomography; CT, computed tomography; EPID, electronic portal imaging device; ETX, ExacTrac X‐ray; MV, megavoltage.

TrueBeam STx in the RT room was equipped with ETX, CBCT, and an electronic portal imaging device (EPID). The Winston–Luz test using the E2E phantom was performed to verify the coincidence between these coordinate systems and the radiation center.^24^ Figure 3 shows the procedure for evaluation of coordinate coincidences between multiple IGRT devices. To acquire images with ETX, infrared markers were placed on the E2E phantom, and a new CT image was scanned. Tube voltage and slice thickness were set to 120 kV and 2 mm, respectively. Automated tube current settings were applied for scanning. The markers were placed asymmetrically to avoid recognition errors. The reference image was reconstructed from the CT image (${\mathrm{Surface}}_{\mathrm{CT}}^{\mathrm{VX}}$), and the center of the E2E phantom was aligned with the isocenter using VOXELAN in the RT room. Under these conditions, CBCT, ETX, and MV images were acquired to evaluate the coincidence of the coordinate origin of the CBCT, ETX, and MV images by EPID based on the phantom sighted at the isocenter by VOXELAN. Since this is a monthly quality assurance procedure, we retrospectively analyzed the data of the last five evaluations from July to October 2023. Incidentally, the VOXELAN was maintained after the second evaluation of the coordinate coincidence.

## 3 RESULTS

### 3.1 Comparison of detection accuracy in different reference image acquisition methods

**FIGURE 4:**
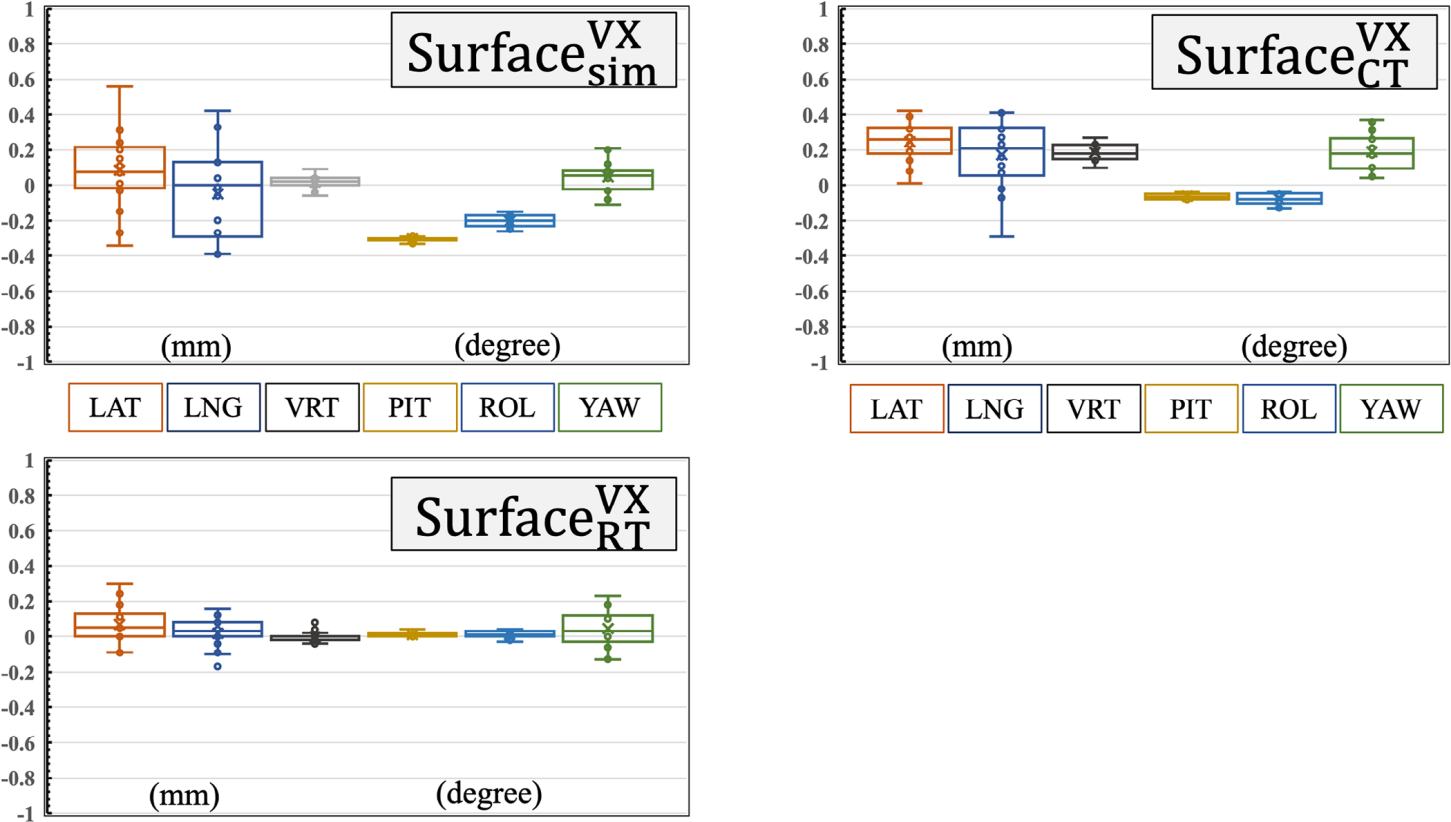
Result of detection accuracy by different reference image acquisition methods. Left upper, right upper, and left bottom figures indicate the detection accuracies of ${\mathrm{Surface}}_{\mathrm{sim}}^{\mathrm{VX}}$, ${\mathrm{Surface}}_{\mathrm{CT}}^{\mathrm{VX}}$, and ${\mathrm{Surface}}_{\mathrm{RT}}^{\mathrm{VX}}$, respectively. LAT: lateral direction, LNG: longitudinal direction, VRT: vertical direction, PIT: pitch angle, ROL: roll angle, YAW: yaw angle.

We assessed the detection accuracy of the three reference image acquisition methods using an E2E phantom. Figure 4 and Table 1 show the results of comparing the detection accuracies of the different reference image acquisition methods. The detection accuracy of ${\mathrm{Surface}}_{\mathrm{sim}}^{\mathrm{VX}}$ in the lateral, longitudinal, and vertical directions and the pitch, roll, and yaw angles were 0.08 ± 0.21 mm, 0.00 ± 0.25 mm, 0.02 ± 0.04 mm, and −0.30 ± 0.01°, −0.20 ± 0.03°, and 0.06 ± 0.08°, respectively. The detection accuracy of ${\mathrm{Surface}}_{\mathrm{CT}}^{\mathrm{VX}}$ in the lateral, longitudinal, and vertical directions and the pitch, roll, and yaw angles were 0.26 ± 0.11 mm, 0.21 ± 0.18 mm, 0.18 ± 0.05 mm, and −0.07 ± 0.01°, −0.08 ± 0.03°, and 0.18 ± 0.11°, respectively. The detection accuracy of ${\mathrm{Surface}}_{\mathrm{RT}}^{\mathrm{VX}}$ in the lateral, longitudinal, and vertical directions and the pitch, roll, and yaw angles were 0.05 ± 0.11 mm, 0.03 ± 0.08 mm, −0.01 ± 0.03 mm, and 0.01 ± 0.01°, 0.01 ± 0.02°, and 0.03 ± 0.01°, respectively. All results are presented as median ± 1 SD, and the maximum value did not exceed 1 mm. Because ${\mathrm{Surface}}_{\mathrm{RT}}^{\mathrm{VX}}$ was the reference obtained in the RT room, theoretically, all values should be zero. However, the mean value for each direction was not zero, and the 1 SD values were larger in the lateral and longitudinal directions.

**TABLE 1:** Result of detection accuracy in different reference image acquisition methods.

		LAT (mm)	LNG (mm)	VRT (mm)	PIT (degree)	ROL (degree)	YAW (degree)
${\mathrm{Surface}}_{\mathrm{sim}}^{\mathrm{VX}}$	Mean	0.09	−0.05	0.02	−0.31	−0.20	0.05
	Median	0.08	0.00	0.02	−0.30	−0.20	0.06
	SD	0.21	0.25	0.04	0.01	0.03	0.08
${\mathrm{Surface}}_{\mathrm{CT}}^{\mathrm{VX}}$	Mean	0.25	0.17	0.19	−0.06	−0.08	0.19
	Median	0.26	0.21	0.18	−0.07	−0.08	0.18
	SD	0.11	0.18	0.05	0.01	0.03	0.11
${\mathrm{Surface}}_{\mathrm{RT}}^{\mathrm{VX}}$	Mean	0.07	0.02	0.00	0.01	0.01	0.04
	Median	0.05	0.03	−0.01	0.01	0.01	0.03
	SD	0.11	0.08	0.03	0.01	0.02	0.10

Abbreviations: LAT, lateral direction; LNG, longitudinal direction; PIT, pitch angle; ROL, roll angle; SD, standard deviation; VRT, vertical direction; YAW, yaw angle.

### 3.2 Evaluation of coordinate coincidence between multiple IGRT devices

**FIGURE 5:**
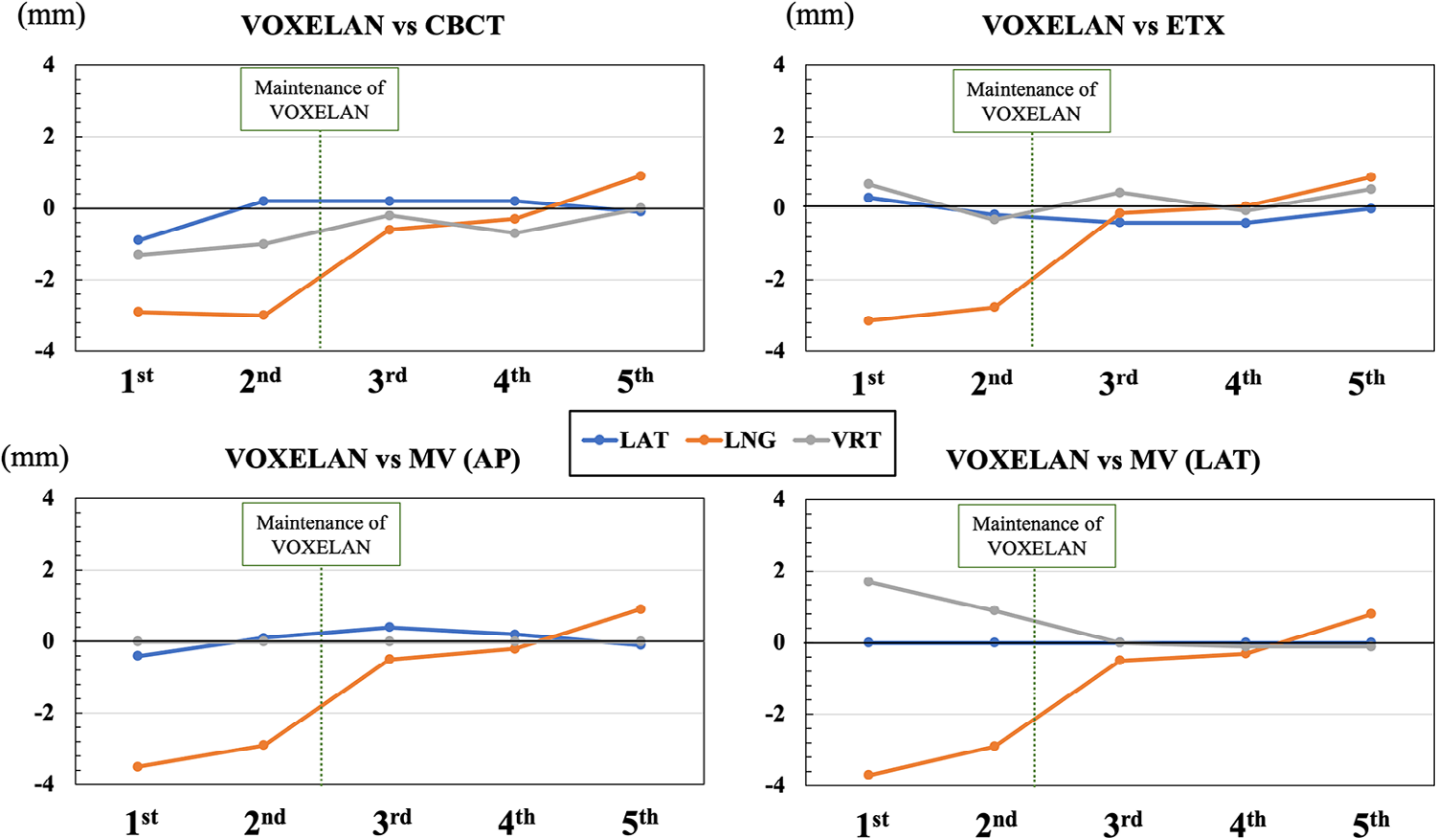
Result of coordinate coincidence between multiple IGRT devices. The upper left, upper right, bottom left, and bottom right figures indicate the coordinate coincidences of VOXELAN versus CBCT, VOXELAN versus ETX, VOXELAN versus MV(AP), and VOXELAN versus MV(LAT) images. The blue, orange, and gray lines indicate the discrepancies in the lateral, longitudinal, and vertical directions, respectively. LAT: lateral direction, LNG: longitudinal direction, VRT: vertical direction.

Figure 5 compares each modality in the last five Winston–Lutz‐based coordinate coincidence tests. The differences observed between the metallic ball and the center of each image modality were illustrated. For that, the E2E phantom was previously setting up to achieve zero displacements detected by VOXELAN. In the first evaluation, the coordinate coincidences between VOXELAN and CBCT in the lateral, longitudinal, and vertical directions were −0.9, −2.9, and −1.3 mm, respectively. The coordinate coincidences between VOXELAN and ETX in the lateral, longitudinal, and vertical directions were 0.29, −3.15, and 0.67 mm, respectively. The coordinate coincidence between the VOXELAN and portal images was less than 1 mm, except in the longitudinal direction (exceed 2 mm at 1st and 2nd evaluation). Based on these results, the camera and the coordinates of VOXELAN in the RT room were calibrated using a calibration phantom. Compared to the first result, the second result slightly improved the lateral and vertical directions for each IGRT configuration. However, coordinate inconsistency in the longitudinal direction did not improve. Therefore, to improve the discrepancy in VOXELAN‐based patient localization, we adjusted the coordinate coincidence between simulation CT and VOXELAN coordinate systems. After comprehensive adjustment of VOXELAN coordinate system that includes VOXELAN in simulation CT room and VOXELAN in treatment room, the coordinate coincidences with other IGRT systems were determined. In the third evaluation performed after comprehensive adjustment, the coordinate coincidences between VOXELAN and CBCT in the lateral, longitudinal, and vertical directions were 0.2, −0.6, and 0.2 mm, respectively. The coordinate coincidences between VOXELAN and ETX in the lateral, longitudinal, and vertical directions were −0.4, −0.14, and 0.44 mm, respectively. In addition, the coordinate coincidence between the VOXELAN and portal image improved within 1 mm owing to the discrepancy in the longitudinal direction. The results showed that the comprehensive coordinate coincidences considering all IGRT devices improved compared to those in the second evaluation. The fourth coordinate coincidence evaluation, conducted afterward, did not exhibit a change as significant as that between the second and third evaluations, and the results were similar to those obtained in the third evaluation. However, in the fifth evaluation of coordinate coincidences, the coordinate coincidence in the longitudinal direction component worsened for all IGRT configurations.

## 4 DISCUSSION

### 4.1 Phantom design

The light‐section method is a technique for non‐contact measurement of the shape or contour of an object or surface when there is an obstacle (usually an object contour) that blocks the light between the line of light emitted from the light source and the camera. The light‐section method is a type of triangulation method. The triangulation method calculates the distance from the camera to the object by identifying a triangle connecting the camera, another device and an object. According to the congruence condition of the triangle, if the distance between the camera and the device is known, the distance between the camera and the object can be calculated as long as the remaining two parameters of the distance or angle between the three parties can be calculated. The light‐section method finds a triangle connecting the camera, the light source and object.

The VOXELAN is an SGRT device that applies the light section method and can be installed in any radiation therapy machine, regardless of the manufacturer. Commercially available quality assurance phantom was developed to evaluate detection accuracy and quality assurance for SGRT devices. The phantoms were designed for applying to other SGRT devices that are based on coded patterned light projections. However, these phantoms cannot be applied to VOXELAN, which is based on the light‐section method. Saito et al. evaluated detection accuracy of setup procedure with VOXELAN.^25^ They used several phantoms including whole body human, head and neck, breast and abdomen shapes to evaluate translational and rotational errors. Their article is one of the few papers on spatial uncertainty using the VOXELAN. However, what they evaluated was the difference between the amounts of known and detected displacements, not the coincidence of coordinate systems. Therefore, in this study, we developed a new E2E phantom compatible with the light‐section method. The shape and material of the phantom were optimally designed for detection using the light‐section method.^22^ Furthermore, VOXELAN can be installed in a CT room to ensure that the positioning conditions during the CT treatment planning can be transmitted to the VOXELAN in the radiotherapy room. In this environment, three reference images can be acquired. However, no phantom has supported the light‐section method for evaluating coordinate coincidence. Because an E2E phantom which contain a metallic ball to allow Winston‐Lutz test can be scanned using CT, it is also possible to create a reference image such as ${\mathrm{Surface}}_{\mathrm{CT}}^{\mathrm{VX}}$. If the upper portion of the plate in this phantom is replaced with one that detected by other patterned light projection SGRT devices, it is possible to perform E2E testing in the same procedure.^8^, ^26^, ^27^, ^28^, ^29^

### 4.2 Comparison of detection accuracy by different reference image acquisition methods

The algorithm of the light‐section‐based method of VOXELAN uses two red lasers to scan an object and determine its three‐dimensional structure.^11^ Therefore, the time difference between the two laser scans and the delay in the reconstruction affected the values. In addition, the differences in the reference data generation methods affected the value. Consequently, the detection data values vary even when the E2E phantom is stationary. Hence, a stationary E2E phantom should be acquired for a certain period, and the raw data should be analyzed to evaluate the change in detection values before clinical implementation. We defined the mean value and standard deviation of the raw data as the detection accuracy and detection stability, respectively. If the detection accuracy and stability differ among the three methods, the reference data cannot be used even if the installation arrangement of the equipment is the same. Therefore, this evaluation should be performed using commissioning data. In the present study, the detection accuracy and detection stability are expressed as median and standard deviation of 21 detection values, respectively. A body surface image detected by VOXELAN in a simulation CT room (${\mathrm{Surface}}_{\mathrm{sim}}^{\mathrm{VX}}$) showed the worst detection stability. In particular, the detection stability in the lateral and longitudinal directions was worse than that of the other acquisition methods.^21^ This is assumed to be the influence of the objects around the E2E phantom during the acquisition of ${\mathrm{Surface}}_{\mathrm{sim}}^{\mathrm{VX}}$ data. Although the installation arrangement of VOXELAN in the simulation CT and the RT room was the same, there was nothing around the isocenter of the radiotherapy device to generate a disturbance shadow. However, a CT gantry has less clearance around the isocenter than TrueBeam, which may interfere with the effective field‐of‐view of VOXELAN in the simulation CT. The fact that both mean and median values appear to be the different for ${\mathrm{Surface}}_{\mathrm{sim}}^{\mathrm{VX}}$ and ${\mathrm{Surface}}_{\mathrm{CT}}^{\mathrm{VX}}$ at all angles may be because of the environment surrounding the isocenters in the CT and RT rooms. To prevent this effect, we tilted the CT gantry to obtain reference data; however, the reflection of the CT gantry in the depth direction remained unchanged. This effect may have caused slight differences in the shapes of the reference data. Because the reference data in ${\mathrm{Surface}}_{\mathrm{CT}}^{\mathrm{VX}}$ were reconstructed from CT images, they were unaffected by the VOXELAN installation arrangement and surrounding isocenter conditions. However, compared to the reference data generated by VOXELAN in the RT room (${\mathrm{Surface}}_{\mathrm{RT}}^{\mathrm{VX}}$), the detection stability in the longitudinal direction was worse than that in the other directions. The detection accuracy between the ${\mathrm{Surface}}_{\mathrm{CT}}^{\mathrm{VX}}$ and ${\mathrm{Surface}}_{\mathrm{RT}}^{\mathrm{VX}}$ data was less than 0.2 mm. This value is negligible because it is smaller than one voxel in the CT image. The detection stability findings showed few differences between the lateral and vertical directions; however, the detection stability in the longitudinal direction was worse. This may be because of the slight difference in shape between the data reconstructed from CT (${\mathrm{Surface}}_{\mathrm{CT}}^{\mathrm{VX}}$) and the data created by VOXELAN in the RT room (${\mathrm{Surface}}_{\mathrm{RT}}^{\mathrm{VX}}$). However, the translational shift and rotational angle errors were less than 1 mm and 1°, respectively. We believe that this value is similar to the positioning accuracy of other SGRT devices shown in the AAPM TG‐302.^8^ Therefore, the SGRT implementation requirements were satisfied for all acquisition methods for the reference data.

### 4.3 Evaluation of coordinate coincidence between multiple IGRT devices

As shown in Figure 5, there was a 2 mm error in longitudinal coordinate coincidence between VOXELAN and other IGRT devices (CBCT, ETX, MV) until the second E2E testing was conducted. Table 2 shows the summary of the daily QA of isocenter check conducted between the 1st and 5th E2E testing. Term 1 to 4 indicate the period between each of the 1st to 5th E2E testing, respectively. As shown in Table 2, isocenter checks of the ETX, CBCT and EPID devices did not show any changes exceeding 2 mm as shown in Figure 5. In this study, CT scan was performed with the E2E phantom placed at the reference position of the simulation CT, and the coordinate coincidence between the ${\mathrm{Surface}}_{\mathrm{CT}}^{\mathrm{VX}}$ and the IGRT device was evaluated. In other words, it is an evaluation of comprehensive coordinate coincidence from the reference coordinates of the CT to the reference coordinates of the IGRT device. The isocenter checks of all IGRT devices showed coincidence within 1 mm, which suggests that there was a discrepancy between the reference coordinates of simulation CT and each IGRT device until the 2nd E2E testing This discrepancy was resolved by carrying out VOXELAN maintenance, including matching the reference coordinates of the simulation CT.

**TABLE 2:** The results of isocenter coincidence daily quality assurance.

		Term 1 (*n* = 25)	Term 2 (*n* = 25)	Term 3 (*n* = 24)	Term 4 (*n* = 25)
ETX	LAT	0.09 ± 0.13	−0.02 ± 0.07	0.18 ± 0.16	0.07 ± 0.18
	LNG	−0.02 ± 0.14	0.09 ± 0.19	−0.16 ± 0.24	−0.18 ± 0.18
	VRT	0.19 ± 0.24	0.02 ± 0.08	0.04 ± 0.09	0.05 ± 0.14
CBCT	LAT	0.06 ± 0.16	0.01 ± 0.12	−0.09 ± 0.17	−0.20 ± 0.12
	LNG	−0.28 ± 0.14	−0.14 ± 0.12	−0.14 ± 0.20	−0.27 ± 0.16
	VRT	0.06 ± 0.14	0.16 ± 0.11	0.24 ± 0.14	0.14 ± 0.23
EPID	LAT	0.10 ± 0.11	0.03 ± 0.08	−0.04 ± 0.11	−0.11 ± 0.11
	LNG	−0.30 ± 0.17	−0.24 ± 0.16	−0.33 ± 0.18	−0.42 ± 0.21
	VRT	0.09 ± 0.11	0.22 ± 0.11	0.26 ± 0.16	0.19 ± 0.23

*Note*: Term 1: between 1st and 2nd E2E testing, Term 2: between 2nd and 3rd E2E testing, Term 3: between 3rd and 4th E2E testing, Term 4: between 4th and 5th E2E testing.

The value means median ± 1SD. The unit of all values is mm.

Abbreviations: CBCT, cone beam computed tomography; EPID, electronic portal imaging device; ETX, ExacTrac X‐ray; LAT, lateral direction; LNG, longitudinal direction; VRT, vertical direction.

Laaksomaa et al. compared the feasibility of IGRT devices using AlignRT, Catalyst, and RPM.^30^ They reported that the SGRT technique improved isocenter reproducibility. However, these results are attributed to the combination of orthogonal IGRT devices. Therefore, the coordinate accuracy between multiple IGRT devices in conjunction with SGRT devices, is important for maintaining the accuracy of the target at the isocenter. The new E2E phantom can evaluate coordinate coincidence based on the Winston–Lutz test, even for light‐section‐based SGRT devices, by targeting a metallic ball in the phantom. In addition, by employing the E2E phantom for quality assurance, it is possible to use this data to adjust the coordinates of the VOXELAN while considering other IGRT devices. Based on the second coordinate coincidence evaluation results, VOXELAN maintenance was performed, and coordinate coincidence was improved in the third evaluation. The most influential component of VOXELAN maintenance was the adjustment of the CT and VOXELAN coordinate systems. Furthermore, the comparison of detection accuracy was evaluated before the adjustment of the CT and VOXELAN coordinate systems. This result implies that the positioning of the reference data and patient's body surface is performed in the VOXELAN coordinate system such that if there is a misalignment between the CT coordinate system (CT scale) and VOXELAN coordinate system (CVX scale), VOXELAN is unable to detect the misalignment. We evaluated this before the clinical implementation of reference data from both the VOXELAN in CT and the reconstructed simulation CT reference data. Therefore, there was no influence on the actual radiation delivery to the patient. However, VOXELAN tends to have a low detection accuracy in the longitudinal direction, which is a characteristic of the system.^11^ Therefore, even if the system was corrected by maintenance, the coordinate coincidence in the longitudinal direction gradually worsened with time since the VOXELAN was maintained. Periodic QA using this E2E phantom is recommended to avoid the effects of longitudinal‐direction inconsistencies.

During deep inspiration breath‐hold (DIBH) breast irradiation, matching the surface detection images in the CT room with the actual CT images is important. In DIBH breast irradiation, it is more important to perform breath‐hold irradiation than achieve an overall uncertainty of less than 1 mm between image‐guidance devices.^31^ Therefore, the SGRT system is valuable as a reference image acquired and used in the RT room rather than reconstructed from CT images. However, when stereotactic radiation therapy is performed using only the SGRT system, an error of more than 1 mm can be problematic; therefore, quality control, such as that used in this study, is important.^32^

## 5 CONCLUSION

We developed a new E2E phantom designated as two hills containing a metallic ball for a light‐section‐based SGRT system. The phantom can also evaluate the coordinate coincidence in multiple image‐guided configurations based on the Winston–Lutz test. We used the developed phantom to compare three different methods for generating reference data: a body surface image detected by VOXELAN in the simulation CT room (${\mathrm{Surface}}_{\mathrm{sim}}^{\mathrm{VX}}$), a body surface image reconstructed from the volume data of the simulation CT (${\mathrm{Surface}}_{\mathrm{CT}}^{\mathrm{VX}}$), and a body surface image acquired by VOXELAN in the RT room (${\mathrm{Surface}}_{\mathrm{RT}}^{\mathrm{VX}}$). This evaluation revealed that ${\mathrm{Surface}}_{\mathrm{sim}}^{\mathrm{VX}}$ and ${\mathrm{Surface}}_{\mathrm{CT}}^{\mathrm{VX}}$ had lower detection accuracy and detection stability than ${\mathrm{Surface}}_{\mathrm{RT}}^{\mathrm{VX}}$. However, the maximum value on ${\mathrm{Surface}}_{\mathrm{sim}}^{\mathrm{VX}}$ and ${\mathrm{Surface}}_{\mathrm{CT}}^{\mathrm{VX}}$ does not exceed 1 mm and 1°. Furthermore, an evaluation of the coordinate coincidences with multiple image‐guidance systems showed that coordinate inconsistencies occurred if the CT coordinate system or other coordinate systems were not adjusted, even if the VOXELAN coordinate system was calibrated. By performing periodic QA and VOXELAN maintenance with the E2E phantom, it is possible to perform light‐section‐based SGRT in a multiple IGRT configuration, not only to confirm the detection accuracy and detection stability but also to ensure that the coordinates of all modalities are coincident.

## AUTHOR CONTRIBUTIONS

All authors contributed to the conception and design of this study. Material preparation, data collection, and analyses were performed by Naoki Hayashi, Tatsunori Saito, and Kazuki Ouchi. Through a review of the results, Hiroshi Amma, Yuta Muraki, Keisuke Yasui, and Masashi Nozue contributed several clinical perspectives to improve this study. The first draft of this manuscript was written by Naoki Hayashi and polished by all the authors. All authors read and approved the final manuscript prior to its submission.

## CONFLICT OF INTEREST STATEMENT

The authors declare no conflicts of interest.

## ETHICS STATEMENT

Ethical approval was not required because this study was a phantom‐based examination.
